# Quantifying motor unit loss prior to functional impairment in muscles affected by amyotrophic lateral sclerosis

**DOI:** 10.1016/j.cnp.2026.06.012

**Published:** 2026-06-30

**Authors:** Boudewijn T.H.M. Sleutjes, Anna Bystrup Jacobsen, Hatice Tankisi, N. Gorkem Sirin, A. Emre Oge, Robert D. Henderson, Pieter A. van Doorn, Leonard H. van den Berg, Ruben P.A. van Eijk

**Affiliations:** a Department of Neurology, Brain Centre Utrecht, University Medical Centre Utrecht, Utrecht, The Netherlands; b Biostatistics and Research Support, Julius Centre for Health Sciences and Primary Care, University Medical Centre Utrecht, Utrecht, The Netherlands; c Department of Clinical Neurophysiology, Aarhus University Hospital, Aarhus, Denmark; d Department of Neurology, Istanbul Faculty of Medicine, Istanbul University, Istanbul, Turkey; e Department of Neurology, Royal Brisbane & Women’s Hospital, Brisbane, Australia; f Department of Neurology, Erasmus Medical Center Rotterdam, Rotterdam, The Netherlands

**Keywords:** Compound muscle action potential scan, Motor unit number estimation, Monitoring disease progression, ALS/MND, Clinical trials

## Abstract

**Objectives:**

The compound muscle action potential (CMAP) scan is a non-invasive method for deriving motor unit number estimates (MUNE) to track disease progression in muscles affected by amyotrophic lateral sclerosis (ALS). It remains to be established whether and how long motor unit loss precedes functional impairment.

**Methods:**

In 56 patients with ALS, we compared the longitudinal trajectories of MUNE derived from thenar CMAP scans, and fine motor function (FMF) using a functional rating scale. Linear and sigmoidal disease trajectories were modelled from which time differences were estimated between these measures to reach their half-maximum scores.

**Results:**

The normalized linear decline per month was 0.02 (95% CI 0.01 to 0.03) for FMF and 0.03 (95% CI 0.03 to 0.04) for MUNE. Half-maximum of FMF was reached after 26.3 months (95% CI 18.9 to 35.1) for the linear model, while MUNE had a shorter time required to reach 50% of its maximum with 13.0 months (95% CI 10.3 to 16.4). The head-to-head comparison between FMF and MUNE showed that MUNE values reached 50% of its maximum 13.1 months (95% CI 7.0–20.8) earlier. Results were similar for sigmoidal disease trajectories.

**Conclusions:**

Simulated disease trajectories of MUNE values derived from CMAP scans in muscles affected by ALS indicated that MUNE may reach 50% of its maximum in approximately 60% of the time compared to functional impairment.

**Significance:**

These explorative findings underscore how neurophysiological measures may be of use for early disease monitoring, with relevance for both care and research settings.

## Highlights

• Motor unit (MU) loss in amyotrophic lateral sclerosis (ALS) may precede symptoms.
• Compound muscle action potential (CMAP) scans can capture this early time window.
• CMAP-scans may provide early biomarkers to timely assess treatment effects in ALS.

## 1 Introduction

Patients with amyotrophic lateral sclerosis (ALS) are characterized by progressive loss of upper and lower motor neurons resulting in ongoing muscle wasting and increasing functional disability. Neurophysiological techniques have the capacity to capture the underlying ALS pathophysiology by providing information on changes in function, size and number of motor units (Donaghy and Pioro, 2024); its degeneration reflecting the primary mechanism driving functional disability. Markers derived from these techniques not only give insights into the underlying disease processes, but may also serve as potential outcome measures in clinical trials (Ahmed et al., 2022; McMackin et al., 2023).

In patients with ALS, prominent downstream pathophysiological disease processes involve progressive motor unit loss and enlarged motor unit sizes due to collateral reinnervation (Bromberg, 1998). The latter could partially explain that, for some time at least, muscle function in affected muscles is fairly preserved. Estimates indicate that 50% or more of the motor unit pool supplying a muscle may already be lost before clinical signs of muscle weakness or wasting become evident (Aggarwal and Nicholson, 2002; Neuwirth et al., 2017). This suggests a substantial time window in which neurophysiological markers have the capacity to detect the disease course while functional impairment scores remain unchanged, which would be of significant value to refine disease monitoring in clinical trials and improve prognostication for care purposes. While prior studies have shown that neurophysiological markers can track progression in limbs without overt clinical signs (Aggarwal and Nicholson, 2002; Neuwirth et al., 2017; Escorcio-Bezerra et al., 2018), translating these insights into a detailed quantitative assessment of the extended early time window remains, however, relatively scarce (Aggarwal and Nicholson, 2002; Neuwirth et al., 2017; Ebersbach et al., 2023). This may be partly because the extent of this early time window depends on multiple factors including the applied neurophysiological technique, functional measure, and also patient characteristics. One of these techniques involves the compound muscle action potential (CMAP) scan. The CMAP scan is a tolerable, broadly available, easy-to-apply technique that provides reliable electrophysiological-derived estimates which can be utilized for capturing the disease process of progressive motor unit loss (Henderson et al., 2007; Baumann et al., 2012; Jacobsen et al., 2019).

A comparison with clinical endpoints would give valuable insights into how much sooner disease progression could be detected with neurophysiological markers. We have shown previously the potential of CMAP scans for monitoring disease progression in ALS clinical trials (Sleutjes et al., 2021), however, a quantitative characterization of the temporal association between motor unit loss and functional impairment was not thoroughly investigated. In this explorative study, we therefore aim to quantify the temporal changes in CMAP scan derived motor unit number estimates relative to functional impairment, assessed by the ALS functional rating scale (ALSFRS-R), a well-established measure in ALS clinical trials.

## 2 Methods

### 2.1 Participant data - electrophysiological and clinical

We used a previously collected multicenter electrophysiological and clinical dataset (Sleutjes et al., 2021). This dataset included longitudinal CMAP scan recordings from the same thenar muscle in each patient with ALSFRS-R scores obtained at each visit. The maximum observed follow-up time in these patients was 15 months. The CMAP scans were either performed on a Viking Select EMG system (Natus Neurology Incorporated, Inc., Middleton, WI, USA) using the CMAP-scan program or with the excitability software (MScan-program, Qtrac-S, institute of Neurology, Queen Square, London, UK). Thenar CMAPs were recorded in belly-tendon configuration using surface electrodes in response to transcutaneous stimulation of the median nerve at the level of the wrist. Stimuli were delivered with gradual reducing stimulus currents from supramaximal to subthreshold levels, yielding recordings with a duration of up to approximately 10 min. We extracted the previously analyzed CMAP scan derived motor unit number estimates (MUNE), which were obtained using the original MScanFit tool (Bostock, 2016) and where we applied the default optimization settings (e.g., each MU had an initial relative spread of 2% and the smallest MU was set at ≥25 μV). In this tool, simulated CMAP scans were generated based on a mathematical model that incorporates variables for MU number, MU sizes and threshold properties, which were automatically optimized to improve the fit between recorded and simulated CMAP scans.

With respect to the subdomains within the ALSFRS-R score, the fine motor function (FMF) subdomain corresponds most closely with hand and arm motor function and therefore yields the best comparison with the available recordings performed in the thenar muscles. As MUNE values showed the highest sensitivity for monitoring disease progression(Sleutjes et al., 2021), we compared MUNE with the FMF subdomain (sum of ALSFRS-R items 4, 5 and 6).

### 2.2 Statistical analysis

We first applied the well-established ALSFRS-R progression rate (Kimura et al., 2006), where at symptom onset we expect the score to be at its maximum, including the FMF subscore (i.e., 12). We then aligned the visits relative to the date of symptom onset. MUNE values at disease onset (i.e. no loss) were set to 105 ± 25 MUs (±SD) to capture normative variability (Song et al., 2022; Stikvoort Garcia et al., 2022), with superimposed a subtle age-dependent decline (Sorensen et al., 2023). Each patient had a lower limit at 20% below the observed maximum MUNE based on the method's reliability expressed by the coefficient of variation (Sorensen et al., 2023). As such, we did not rely on a single reconstructed baseline MUNE value per patient but incorporated inter-individual variability while accounting for patient-specific uncertainty. Additionally, rather than assuming no MU loss before symptom onset, treating MU loss as temporally fixed and identical across patients, we conducted a sensitivity analysis in which MU loss was assumed to occur predominantly shortly before symptom onset. This was modelled using an exponential-distributed interval relative to symptom duration to account for increasing uncertainty associated with recall bias (2SD 5% of symptom duration). As a result, we had 3 to 8 longitudinal samples for each participant (median of 4), including the simulated baseline FMF scores and MUNE values. To further facilitate comparison, we normalized MUNE and FMF per patient.

**Fig. 1:**
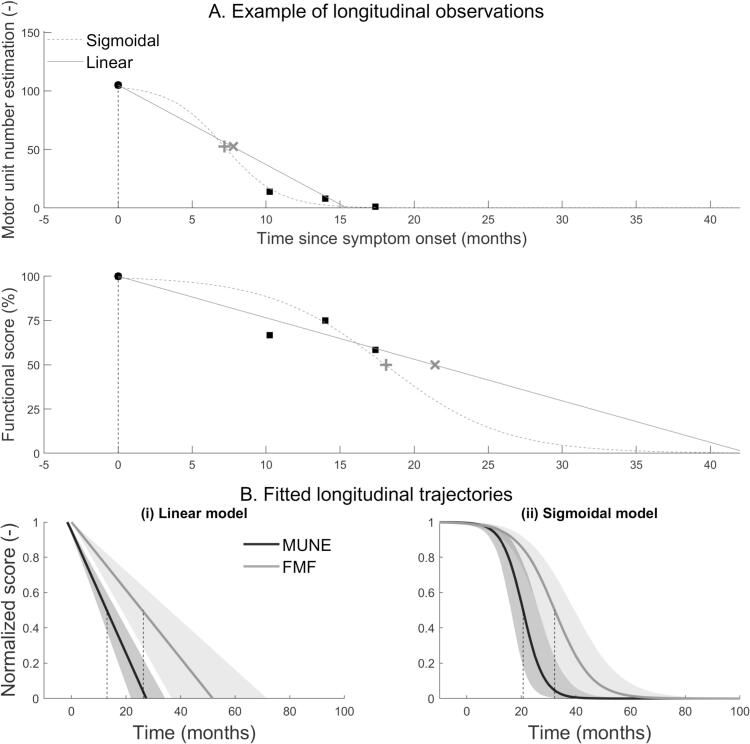
(A) Illustrative example of MUNE (top) and normalized FMF (bottom) observations (black squares) and their simulated baseline values (black circles). It shows the estimated time to reach 50% of their maximum for the linear (crosses) and sigmoidal (plusses) trajectories. MUNE compared to the functional score reached 50% of its maximum earlier for the linear (21.4 vs. 7.8 months) and sigmoidal (18.1 vs 7.2 months) model. Note that baseline MUNE was set to 105 (vertical dashed) at x = 0 reflecting date of symptom onset, whereas these values varied in the simulations for sensitivity analysis. This example reflects an individual patient, while the primary analysis is on population level. (B) The normalized longitudinal trajectories for MUNE (dark gray) and FMF (light gray) using (i) the linear and (ii) sigmoidal model (solid lines show median progression; shaded areas the bootstrapped 95% confidence interval). The vertical dotted lines represent on population level the median time to reach 50% of their maximum (MUNE vs FMF – (i) 13.0 vs 26.3 months – linear; (ii) 20.7 vs 32.2 months - sigmoidal).

We then fitted longitudinal trajectories with mixed effects models (*nlmefit*-function, Statistics and Machine Learning Toolbox, Matlab R2023a, Wisconsin). Model parameters were estimated using maximum likelihood and goodness-of-fit measures included Akaiki information criterion and root mean square error of the residuals. Advantageously, mixed effects models incorporate all CMAP-scan recordings from the entire patient cohort, allowing us to use the entire longitudinal dataset rather than restricting the analysis to individual observed recordings only. It can further handle unequal visits and time intervals between individual patients. We, first, modelled a frequently applied linear disease course with a fixed effect of time since onset in months, and a random slope for time per patient. We also applied a sigmoidal disease course (Ebersbach et al., 2023) using fixed effects describing time since onset in months (D50) and slope where the curve reaches 50% of its maximum, and a random effect of time at this point per patient. To aid convergence, the slope was modelled as a function of time to reach 50% of its maximum given their strong correlation (Poesen et al., 2017). We used bootstrapping (*N* = 1000) to calculate the 95% CI of the longitudinal declines and absolute time difference to reach 50% of its maximum between FMF and MUNE. To quantify the time difference, we used the estimated difference of the fixed effects for the time to reach 50% of its maximum for the linear and sigmoidal model. We applied both models on our dataset as a sensitivity analysis to evaluate the impact of their assumptions and to confirm that the estimated time differences were robust to the choice of the modelled disease trajectory models. A schematic view of the analysis is illustrated in Fig. 1A.

## 3 Results

We had CMAP scan data and ALSFRS-R and FMF scores available from 56 patients with MND. These 56 patients included patients with ALS (*n* = 45) and PMA (*n* = 11) with a median age of 65 years (range 28–83) of whom 18 (32%) were female. As ALS and PMA are considered to share a common clinical spectrum (Kim et al., 2009), we grouped them together for further analysis, which is also in line with the latest consensus criteria (Kiernan et al., 2020). Of the 56 patients, symptom onset was bulbar in 16 patients, upper limb in 19 patients and lower limb in 23 patients. The median disease duration was 12.6 months (range 2.8–120). The median ALSFRS-R was 41 (range 9–48) with a median FMF subscore of 10 (range 0–12) at first visit. A substantial proportion of patients had an initial observed FMF with a maximum score of 12 (*n* = 16) indicating yet unaffected functional scores in this domain. In these 56 patients, there were 182 CMAP scans available and after a visual quality check (e.g., excessive (in)voluntary activity, movement artifacts or noise), all were retained for analysis. The ALSFRS-R had a median D50 of 38.9 months (95% CI, 33.5 to 44.3; *n* = 56), and slope, was 9.8 months (95% CI, 7.9 to 12.0; n = 56), which is comparable to findings in previous studies (Poesen et al., 2017; Ebersbach et al., 2023).

When comparing FMF with MUNE, the mean normalized monthly linear decline was 0.02 (95% CI, 0.01 to 0.03) for FMF and 0.03 (95% CI, 0.03 to 0.04) for MUNE. For the FMF score, the time to reach 50% of its maximum was 26.3 months (95% CI 18.9 to 35.1; linear) and 32.2 months (95% CI, 26.2 to 39.4; sigmoidal). Compared to the FMF subdomain, the longitudinal trajectories for MUNE showed a shorter time required to reach 50% of its maximum for both models (linear – 13.0 months, 95% CI 10.3 to 16.4; sigmoidal – 20.7 months, 95% CI, 16.2 to 26.3). The head-to-head comparisons (Fig. 1B) between FMF and MUNE showed that MUNE reached 50% of its maximum earlier with on average 13.1 months (95% CI 7.0–20.8; linear) and 11.5 months (95% CI 8.2–15.2; sigmoidal). Dividing the time to reach 50% of its maximum of MUNE by FMF yielded 0.62 (95% CI, 0.40 to 0.76) for both models, indicating that it took approximately 60% of the time for MUNE compared to reach 50% of its maximum compared to the time it took for FMF.

## 4 Discussion

In this explorative study, we have provided a quantitative estimate on how much sooner motor unit loss can be detected compared to onset of progressive functional decline within a muscle among patients with ALS. -Our findings indicated that motor unit loss precedes the manifestation of functional impairment by, on average, one year. This time window may offer unique possibilities to enhance monitoring in clinical trials (Neuwirth et al., 2017) and prognostication. This time window may facilitate earlier identification of treatment-induced changes on disease progression as a monitoring biomarker, potentially allowing shorter follow-up in clinical trials. When reflecting changes in underlying disease activity, it may also provide early indications of subsequent functional decline. Generally, insensitive biomarkers for monitoring limits drug development (Katyal and Govindarajan, 2017), whereas earlier disease detection enables timely diagnosis and facilitates enrolment of patients into clinical trials at early disease stages.

Our findings are in support with previous neurophysiological studies (Aggarwal and Nicholson, 2002; Neuwirth et al., 2017; Ebersbach et al., 2023) and aim to encourage further investigation into electrophysiological biomarkers, potentially widening the sensitivity window for monitoring the effects of disease-modifying treatment effects in ALS. The clinical heterogeneity observed in patients with ALS, including variations in disease progression, site of symptom onset, and spread of symptoms, affects the time lag between functional decline and neurophysiological techniques to capture early motor unit loss. In contrast to previous studies, we implemented a more rigorous approach by accounting for uncertainties in the disease trajectory, dates of symptom onset and normative motor unit number values. A targeted measure of muscle strength in the examined muscle would be preferable, since the FMF subdomain is a composite functional measure involving arm function and strength originating from bilateral muscle groups and multiple pathways for motor control. However, this would imply greater sensitivity for detecting early functional changes than a more localized neurophysiological measure, which was not observed in our study. The observed time difference between FMF and MUNE may therefore be multifactorial, of which the underlying biological processes and potential differences in scale sensitivity are most likely. As we relied on several model assumptions and given the large confidence intervals for the estimated time window there is need for caution and further design of prospective studies.

In our study, we used common linear and sigmoidal frameworks for modelling disease trajectories, although alternative (non)linear frameworks (Thakore et al., 2018) should be considered for comparing disease trajectories of multiple measures, which would better suited using larger and diverse clinical and neurophysiological datasets. We further focused on assessing the gain in time at early disease stages, indicating the reduced sensitivity for functional score due to potential ceiling effect in monitoring disease progression. Conversely, at later disease stages, the likelihood of undetectable motor unit loss in severely wasted muscles poses other challenges (‘floor effects’). We therefore acknowledge the limitations of the current neurophysiological dataset obtained from a single muscle (Sleutjes et al., 2021) (i.e., APB), which is also known to be affected early, and therefore may have led to bias towards detecting these early changes. Sampling more muscles may eventually provide a more generalized view on this early time window on patient level. Due to a relatively small number of visits per patient, we relied on reconstructed baseline MUNE values for which we used a comprehensive multicenter normative dataset (Sorensen et al., 2023). We therefore also restricted our primary analysis to population-level effects, while individual trajectories will eventually be more informative, particularly given the disease heterogeneity in ALS. Consequently, given the retrospective design, our explorative findings warrant applying neurophysiological methods in multiple muscles in a larger prospective patient cohort with frequent longitudinal assessments, also including other measures (e.g., muscle strength using dynamometry) for enhanced comparison. Given the marked heterogeneity of ALS, this would also require the collection of more detailed patient-specific information for in-depth phenotyping (e.g., upper motor neuron burden, cognitive involvement, respiratory status, or genetic background). Neurophysiological measures derived from the CMAP-scan primarily assess lower motor neuron degeneration. Therefore, in the end, multimodal characterization, including biomarkers for detecting upper and lower motor loss and dysfunction will ultimately create the possibility to uncover the temporal cascade of changes and dysfunction along the neural axis allowing more in-depth phenotyping of patients with ALS and/or people at risk for developing ALS (Benatar and Wuu, 2012).

When monitoring patients with ALS, neurophysiological measures, including CMAP-scan derived MUNE values, may be a useful biomarker of early motor unit loss before functional impairments are clinically evident. This will require confirmation in a larger cohort and across multiple muscles, potentially contributing to more efficient evaluation of disease-modifying therapies. Rapid changes in multiple biomarkers with varying sensitivities may indicate a more aggressive disease course and poorer prognosis. Ultimately, this approach may expedite the search for sensitive composite biomarkers to timely assess treatment effects for patients with ALS.

## Declaration of competing interest

The authors declare that they have no known competing financial interests or personal relationships that could have appeared to influence the work reported in this paper.
